# The Care of the Skin

**Published:** 1890-09

**Authors:** 


*The Care of the Skin.* By F. AUGUSTUS COX, M.B. London: ALEXANDER & SHEPHEARD. 1890.

This is a thin book of about 50 pages, with pleasantly-written accounts of various kinds of baths, and with some wise and timely observations on the use and abuse of soap, of which, it is a comfort to find, no special maker is named. The shallow chapter on "Diet" might well have been omitted.

